# Relationship between the Severity of Inflammatory Changes in Chronic Sinusitis and the Level of Vitamin D before and after the FESS Procedure

**DOI:** 10.3390/jcm10132836

**Published:** 2021-06-27

**Authors:** Paulina Kalińczak-Górna, Kamil Radajewski, Paweł Burduk

**Affiliations:** 1 Department of Otolaryngology, Phoniatrics and Audiology, Collegium Medicum, Nicolaus Copernicus University, 85-168 Bydgoszcz, Poland; pburduk@wp.pl; 2 Department of Otolaryngology, Laryngological Oncology and Maxillofacial Surgery, University Hospital, 85-168 Bydgoszcz, Poland; kamil.radajewski@gmail.com

**Keywords:** chronic sinusitis, inflammation, vitamin D, FESS procedure

## Abstract

There have been a few reports confirming that vitamin D (VD3) deficiency increases inflammation in the paranasal sinuses. The work brings new information that, despite the presence of inflammation before surgery, patients with higher vitamin D levels had less inflammation, and this has been proven on three levels. We show that vitamin D levels clearly correlate with the severity of the disease in chronic sinusitis. These results have been confirmed in imaging studies (Lund MacKay scale), endoscopy (Lund-Kennedy scale) and in the SNOT 20 questionnaire. 40 patients suffering from chronic sinusitis were divided into two equal groups: group 1: with less severe radiological changes (10 or less points on the Lund-Mackay scale), group 2: with a more advanced form (>10 points). The relationship between VD3 and the severity of the disease (clinical and nasal endoscopy) was assessed. The mean VD3 level in patients in group 2 before surgery was lower (23.01 ng/mL) than in group 1 (28.02 ng/mL) (*p* < 0.05). They presented a higher degree of advanced changes in all the above scales, i.e., before the SNOT 20 procedure, the mean was: group 1: 30.33, group 2: 31.80 (*p* < NS); Lund-Kennedy: group 1: 3.21, group 2: 6.30 (*p* < 0.05). After surgery an increase in VD3 levels was observed in both study groups: in group 1 to the value of 37.98 ng/mL (*p* < 0.002) and in group 2 to 27.67 ng/mL (*p* < 0.004). Lower VD3 levels were found in patients with a higher stage of the disease. Reduction of inflammation increases the level of VD3 and reduces subjective and objective symptoms of chronic inflammation.

## 1. Introduction

Chronic sinusitis (CRS) affects 5–12% of the population [1]. According to the EPOS 2020 guidelines, it is characterized by at least two symptoms, one of which should be either nasal blockage/obstruction/congestion or nasal discharge (anterior/posterior nasal drip). Other symptoms include pain or a feeling of pressure in the face, and a reduction or loss of smell. Among the symptoms visible in nasal endoscopy, we can distinguish:

- presence of nasal polyps and/or
- mucopurulent discharge (especially in the middle nasal meatus) and/or
- swelling of the mucosa (especially in the middle nasal meatus).

Chronic sinusitis is a complex disease with an undetermined etiology. Disorders of the muco-ciliary transport, allergy, asthma, allergy to aspirin, smoking, and genetic factors contribute to the development of the disease [2]. Patients suffering from CRS often have a worse quality of life and social functioning [3], and the need for reoperation negatively affects public health, increasing the costs of treatment. There are many publications confirming that chronic inflammatory diseases are associated with reduction in vitamin D levels [4,5,6,7,8]. Its deficiency increases the inflammation and promotes the occurrence of chronic paranasal sinusitis [9,10]. Removal of inflammation during the FESS (functional endoscopic sinus surgery) procedure increases the value of vitamin D, reduces inflammatory markers, and improves the patient’s quality of life [11,12]. Research suggests that vitamin D3 acts as a steroid hormone that has anti-inflammatory effects and plays an important role in regulating dendritic cell function. Vitamin D3 is able to stop cytokine production and inhibit differentiation of immune cells. This effect inhibits the progression of the disease [13]. The aim of the study was to assess the relationship between serum vitamin D levels and the severity of inflammatory lesions in the paranasal sinuses before and after surgery. There are very few similar studies in the literature, so further research is needed to investigate the above relationships.

## 2. Materials and Methods

The research was carried out on 40 patients suffering from chronic paranasal sinusitis requiring operation. The patients had not been operated on before. A statistical analysis of the size of the test sample was carried out in order to determine the significance at the level of *p* < 0.05 and the test power for 0.80 using the Statistica version 13.1 program (Cracow, Poland). A value of *p* < 0.05 was considered as statistically significant. The study protocol was approved by the local ethics committee (approval number: KB 138/2019). This examination was conducted in accordance with the Helsinki Declaration.

**Figure 1:**
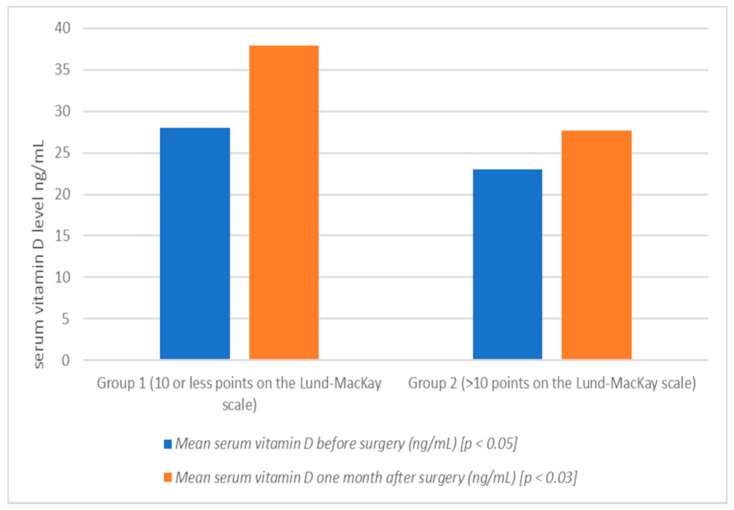
Mean serum vitamin D before and one month after surgery (ng/mL) in group 1 and 2 (graph).

The patients were divided into two groups (Table 1): group 1 (20 patients), patients with less severe radiological changes (<10 points on the Lund-Mackay scale), group 2 (20 patients), patients with a more advanced form of chronic sinusitis (>10 points in the above scale.). Blood samples for the assessment of vitamin D levels were collected after admission to the hospital, and also 1 month after the procedure (Table 2, Figure 1). Vitamin D deficiency was defined as less than 20 ng/mL. During the qualifying visit, the stage of the disease was assessed in patients using CT scan of the sinuses (Lund and MacKay scale), as well as endoscopy (Lund-Kennedy scale). After admission to the hospital, as well as one month after surgery, the endoscopic evaluation was performed again (Lund-Kennedy). Moreover, the patients were assessed for quality of life in chronic sinusitis using the SNOT-20 questionnaire (The Sinonasal Outcome Test).

The exclusion criteria to the study were: patients with other systemic diseases (metabolic diseases, cardiovascular diseases), neoplasms, previous surgical procedures in the period of 6 months before enrollment in the study, and constant or periodic supplementation with vitamin D3 or combined preparations. The study groups were comparable in terms of sex and age.

## 3. Results

**Figure 2:**
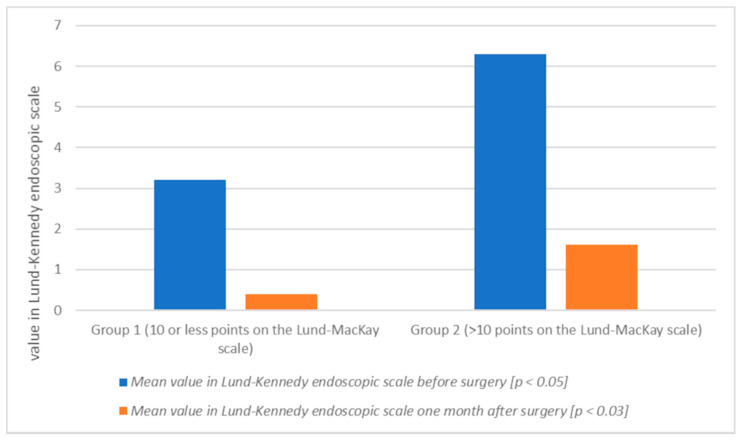
Mean value in Lund-Kennedy endoscopic scale before and one month after surgery in group 1 and 2 (graph).

**Figure 3:**
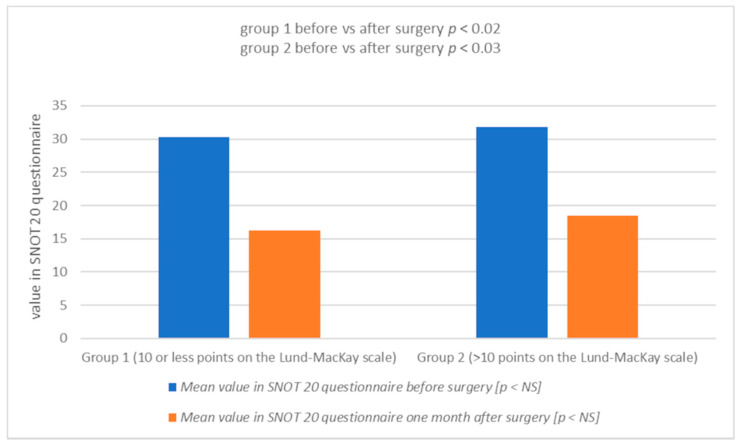
Mean value in the Sinonasal Outcome Test SNOT 20 questionnaire before and one month after surgery in group 1 and 2 (graph; NS: not significant).

The mean serum vitamin D level in patients in group 2 before surgery was lower (23.01 ng/mL) than in group 1 (28.02 ng/mL) (*p* < 0.05) (Table 2, Figure 1). These patients also presented a higher degree of advancement of changes in all the above scales, i.e., before the Lund-Kennedy procedure: group 1: 3.21, group 2: 6.30 (*p* < 0.05) (Table 3, Figure 2); SNOT 20 mean: group 1: 30.33, group 2: 31.80 (Table 4, Figure 3). One month after the surgery, an increase in vitamin D levels was observed in both study groups: in group 1, on average, to the value of 37.98 ng/mL (*p* < 0.002); and in group 2: 27.67 ng/mL (*p* < 0.004) (Table 2, Figure 1). Patients with lower vitamin D levels show a higher degree of severity of changes in the Lund-MacKay, Lund-Kennedy scale and in the SNOT 20 questionnaire (Figure 1, Figure 2 and Figure 3).

## 4. Discussion

Vitamin D (VD3) deficiency is a global problem. Obesity, deficiency of physical activity and less time spent outdoors have a negative effect on its level. It is well known that the origin and climate we live in have an influence on the level of vitamin D. It is higher in countries with more sunny days. The study was conducted on patients living in a temperate climate. Vitamin D has antibacterial, anti-inflammatory and anti-proliferative properties. Low vitamin D levels are associated with a dysfunction of the natural mechanisms that limit the mucositis, proliferation and angiogenesis associated with chronic sinusitis [14].

The effect of vitamin D on the metabolism of serum calcium and phosphate as well as bone formation and resorption processes has been known for a long time, but in recent years it has also been assigned a role in protection against inflammation and autoimmune diseases [15]. It has been shown that decreased serum vitamin D levels, as well as polymorphism of genes encoding enzymes involved in its metabolism, are associated with a higher incidence of asthma, atherosclerosis and multiple sclerosis [4,5,6].

VD3 maintains skin integrity, reduces its colonization by pathogens and suppresses the allergic reaction in atopic dermatitis [7]. A significant association was also found between low VD3 levels in patients with allergic rhino-conjunctivitis, compared to healthy subjects [8].

VD3 plays an important role in the functioning of the respiratory system. VD3 deficiency is inversely correlated with upper respiratory tract infections [11,12]. Optimal levels of VD3 are associated with reduced potential for complications in people with asthma, as well as reduced use of anti-inflammatory drugs [9].

The relationship between vitamin D levels and chronic sinusitis is being increasingly investigated. It has been shown that the epithelial cells of the paranasal sinuses affect the expression of 1-alpha-hydroxylase, and the level of this enzyme is reduced in patients with chronic inflammatory disease [16]. Another study found that the expression of 1alpha-hydroxylase is lower in patients with a higher degree of progression of inflammatory lesions in the CT scan (Lund-MacKay scale), and in patients scoring more points on the SNOT-22 scale reflecting more severe symptoms of chronic sinusitis [17]. In the study we found that higher status of inflammation process in sinuses (higher values in group 2 in scales: Lund-MacKay and Lund-Kennedy) resulted in lower level of serum vitamin D3 and slower healing process (higher values in group 2 in Lund-Kennedy’s scale one month after surgery). Nevertheless, after surgery the serum level of vitamin D3 showed increase in both groups, higher among patients with less severe disease according to Lund-MacKay scale. The initiation of treatment causes the conversion of 25-vit D3 to 1.25-vitamin D3 and the production of the antibacterial peptide, cathelicidin. VD3 acts as an inhibitory factor in the acute phase reaction, and inflammation present from CRS may reduce VD3 levels [18]. Low levels of VD3 in the serum are more common in patients with chronic sinusitis, especially those with polyps (CRSwNP), and in those with eosinophilic (or allergic) chronic sinusitis [18]. A similar relationship in patients with nasal polyps was observed in our study (Table 5). Patients with polyps had significantly reduced levels of vitamin D3 before treatment as well as levels after surgery with lower values. In endoscopic observation, this correlated with a prolonged healing process.

It has been proved that Th2 cells bound to IL-4, IL-5 and IL-13 play the main role in the form of chronic sinusitis with polyps [14]. Vitamin D has anti-inflammatory and immunomodulatory properties, especially in terms of its influence on dendritic cells, T cells and macrophages [1]. In addition, it prevents the maturation and differentiation of monocytes into dendritic cells, and increases the synthesis of interleukin 10, which produces regulatory T cells that reduce inflammation. In further studies it was found that VD3 analogs (calcitriol and tacalcitol) reduce the secretion of RANTES and eotaxin in fibroblast cultures from nasal polyps, and also reduce their proliferation [15,19].

Several cross-sectional studies have assessed computed tomography (CT) severity scores and VD3 levels in patients with CRS [11,12]. A significant relationship was found between decreased VD3 levels and increased bone remodeling in CT [11,12]. Based on the Lund-Mackay scale, a higher degree of disease advancement was observed in patients with lower VD3 levels [11]. The same results were observed in our study. Two studies analyzed the effect of serum VD3 levels on systemic markers of inflammation [9,12]. Lower VD3 levels have been shown to be associated with significantly increased CD209 expression of dendritic cells, elevation of prostaglandin E2 and granulocyte-macrophage colony stimulating factor, all of which are systemic inflammatory markers [13]. It has been proven in in-vitro studies that the active form of vitamin D (1.25 (OH) D3) reduces the production of pro-inflammatory cytokines, i.e., IL6, IL8, RANTES, and eotaxins by the epithelial cells of the paranasal sinuses [17].

Some researchers have analyzed the effects of vitamin D supplementation in patients with chronic sinusitis. They showed that the intake of 4000 IU of vitamin D for a minimum of 4 weeks reduces the symptoms of the disease [10]. Supplementation with the above dose also reduces the frequency of polyp recurrences after FESS surgery [1]. In patients with severe inflammation, in whom high inflammatory parameters and low vitamin D levels were initially observed, they decreased after receiving vitamin D supplements [10]. In our study, we observed significantly lower levels of vitamin D3 in more advanced inflammation stages, which proves that vitamin D is used to reduce inflammation. Both the better healing process and the restoration of natural vitamin D3 resources were faster in patients with less inflammatory changes compared to the group with more advanced inflammation.

## 5. Conclusions

Lower serum vitamin D levels were found in patients with a higher stage of the disease process. Removal of inflammatory changes in the paranasal sinuses increases the level of vitamin D in the serum and helps to rebuild natural resources. More research is needed to establish guidelines for vitamin D supplementation in patients with chronic sinusitis.

## Figures and Tables

**Table 1:** Mean value in Lund-MacKay scale in group 1 and 2.

Group of Patients	Mean Value in Lund-MacKay Scale
1	
(10 or less points on the Lund-Mackay scale)	5.60
2	
(>10 points on the Lund-Mackay scale)	13.33

**Table 2:** Mean serum vitamin D before and one month after surgery (ng/mL) in group 1 and 2.

Group of Patients	Mean Serum Vitamin D before Surgery (ng/mL) [*p* < 0.05]	Mean Serum Vitamin D One Month after Surgery (ng/mL) [*p* < 0.03]
1		
(10 or less points on the Lund-Mackay scale)	28.02	37.98
2		
(>10 points on the Lund-Mackay scale)	23.01	27.67

**Table 3:** Mean value in Lund-Kennedy endoscopic scale before and one month after surgery in group 1 and 2.

Group of Patients	Mean Value in Lund-Kennedy Endoscopic Scale before Surgery [*p* < 0.05]	Mean Value in Lund-Kennedy Endoscopic Scale One Month after Surgery [*p* < 0.03]
1		
(10 or less points on the Lund-Mackay scale)	3.21	0.41
2		
(>10 points on the Lund-Mackay scale)	6.30	1.61

**Table 4:** Mean value in the Sinonasal Outcome Test SNOT 20 questionnaire before and one month after surgery in group 1 and 2.

Group of Patients	Mean Value in SNOT 20 Questionnaire before Surgery	Mean Value in SNOT 20 Questionnaire One Month after Surgery
1		
(10 or less points on the Lund-Mackay scale)	30.33	16.20
2		
(>10 points on the Lund-Mackay scale)	31.80	18.53

**Table 5:** Mean serum vitamin D before and one month after surgery (ng/mL) in patients suffering for chronic sinusitis with nasal polyps (CRSwNP) and those without nasal polyps (CRSsNP).

Groups of Patients with CRS	Mean Serum Vitamin D before Surgery (ng/mL)	Mean Serum Vitamin D after Surgery (ng/mL)
CRSwNP	20.36	24.95
CRSsNP	27.68	31.05

## Data Availability

No public database has been created. All data are available from the authors of the work.
